# Comparative Study of Carbon Materials Synthesized “Greenly” for 2-CP Removal

**DOI:** 10.1038/srep29167

**Published:** 2016-07-04

**Authors:** Ying Ma, Nan Lu, Ying Lu, Jiu-nian Guan, Jiao Qu, Hai-yang Liu, Qiao Cong, Xing Yuan

**Affiliations:** 1 School of Environment, Northeast Normal University, Changchun, Jilin 130024, China; 2 Key Laboratory of Industrial Ecology and Environmental Engineering, Ministry of Education, School of Environmental Science and Technology, Dalian University of Technology, Dalian, Liaoning 116024, China

## Abstract

Carbon nanotubes (CNTs), graphene (GA) and carbon nanospheres (CNSs) were prepared respectively using grass (*Festucaarundinace*) as the sole carbon resource by solvothermal method and characterized as adsorbent and photocatalyst for 2-chlorophenol (2-CP) removal in water. With H_2_O_2_/HNO_3_/H_2_SO_4_, the CNTs were firstly produced from grass (*Festucaarundinace*) at 300 °C by hydrolysis and oxidization, the CNTs were secondly opened to form the GA by oxidization at 400 °C, and the GA was lastly rolled-up to form the CNSs by oxidization at 500 °C. All adsorption equilibration of the CNTs, GA, and CNSs for 2-CP were achieved within 120 min, and 60.35%, 20.12%, and 76.22% of 2-CP (5 mg L^−1^, pH = 6.3) were adsorbed, respectively. Furthermore, the high removal rates of 2-CP were about 88.23%, 92.90%, and 79.64% by the CNTs, GA, and CNSs, after 120 min adsorption and 160 min irradiation. On the basis of these results, the CNSs were suitable for removal 2-CP as adsorbent, and the GA was suitable as photocatalyst. The photooxidation of 2-CP was mainly initiated by O_2_^·−^ or ·OH which was generated from the combine with simulated sunlight and the CNTs or GA, respectively. However, the CNTs was not suitable for removal 2-CP owing to the increasing toxicity.

Monochlorophenols, identified as moderately toxicities for aquatic organism and human even at low concentration (parts per billion), are listed by the EU and USEPA^1^,^2^ as priority pollutants. Among these, 2-chlorophenol (2-CP) is applied as disinfectant/germicide/pesticide, with the properties of slow biodegradability, high solubility and toxicity in water. Chlorination of public water supply can also lead to the generation of 2-CP in drinking water. The maximum allowable threshold of 2-CP is no more than 1 mg L^−1^ in aqueous solution for human consumption^3^. Hence, it is extremely urgent to research and develop the feasible technologies for 2-CP removals in aqueous solution.

In recent years, many technologies have been applied to remove chlorophenols, such as physic-chemical, photo-chemical, and microbiological methods^4^,^5^,^6^, among which adsorption and photocatalytic degradation by adsorbents and catalysts seem to be quite feasible and effective way for contaminate removal^7^,^8^,^9^,^10^. Carbon nanomaterials (CNMs) such as carbon nanotubes (CNTs), graphene (GA) and carbon nanospheres (CNSs) have attracted increasing interest of environmental researcher owing to their adsorptive and optical properties^11^. The specific surface areas of the CNTs, GA, and CNSs were reported to be 86.7–1531.5 m^2^ g^−1 ^12,13, 270–1628 m^2 ^g^−1 ^14,15, and 294.32–2130 m^2 ^g^−1 ^16,17, respectively. The band gaps of the CNTs and GA were 3.1 18 and 1.7 eV^19^. Hence, they are used as adsorbent, carrying agent, and photocatalyst for pollutants removal in environment. Although the cost of the CNMs is decreasing, their current pricing is still a key factor prohibiting its application in the field of environmental protection.

To date, of the various methods available for the CNMs preparation, high energy consumption and using non-renewable raw material are the main weakness of these approaches. Thus, finding low cost and green synthesis methods for preparation of the CNMs would potentially benefit environment. In previous studies, some renewable resources (such as fiber and starch of vascular plant species) were used for preparation of the CNMs without catalyst and through low temperature process^20^,^21^,^22^. However, the chemical compositions of the cell walls play an important role in the preparation of the CNMs. Besides, it is always necessary to employ the multiple cycles (more than 20 times) of heating process (over 400 °C) with oxygen and the degreasing pretreatment to prepare for the CNTs. During photosynthesis, plants transform carbon dioxide into sugars and other carbon-based molecules. The carbon in waste plants always changes to carbon oxides in the treatment and disposal after being harvested and death. Synthesis of the CNMs using vascular plant species as raw material can provide a new sight in carbon recycling in waste plants and further reducing carbon dioxide emissions.

Herein, we report that the CNTs, GA, and CNSs can be prepared respectively using the same grass as the sole carbon resource, without catalyst, and through a simple solvothermal process, which is of great importance for the “green synthesis” of the CNMs and not reported previously to the best of our knowledge. As for the removal of 2-CP in aqueous solution, it is foreseeable that the prepared CNTs, GA, and CNSs have different adsorption capacities and photocatalytic activities. Meanwhile, synthetic routes for three types of the CNMs and mechanisms of removal of 2-CP by them were analyzed and compared in the present work.

## Results and Discussion

### Structure and Morphology of CNTs

**Figure 1:**
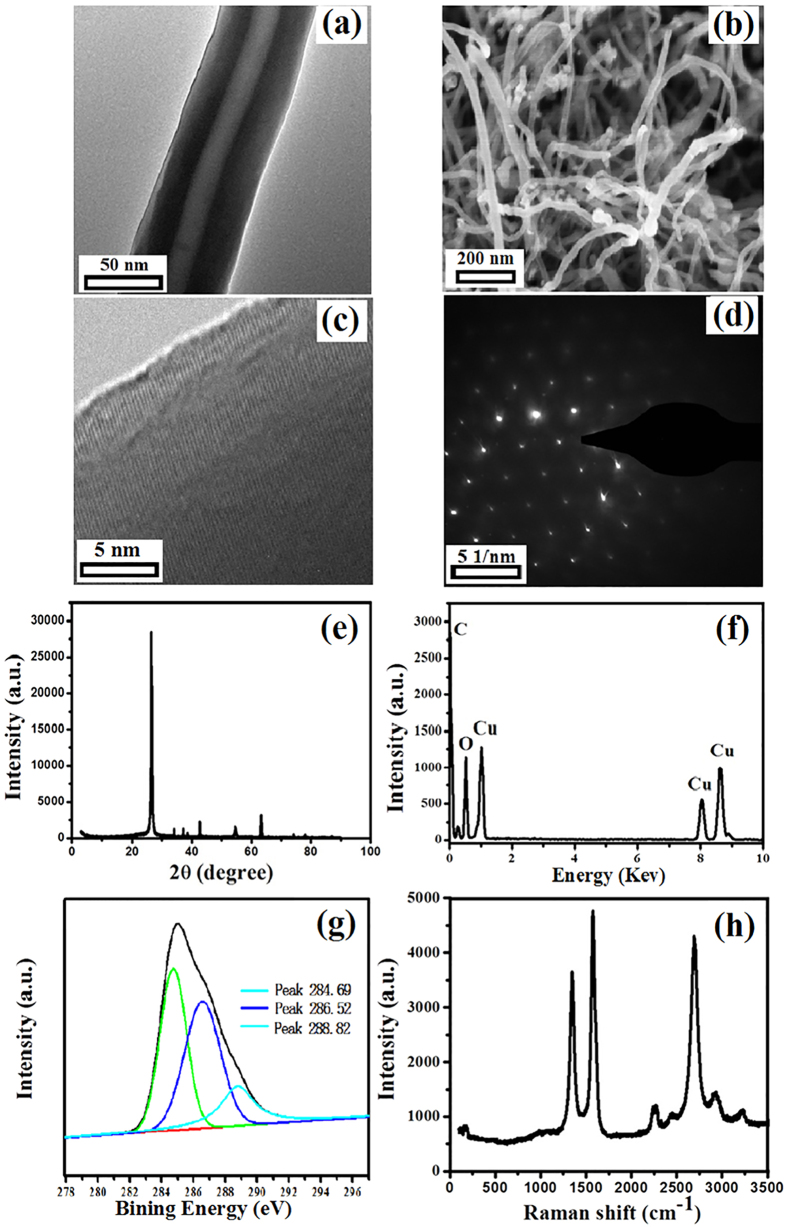
Characterization of the prepared CNTs from grasses: (**a**) TEM image, (**b**) SEM image, (**c**) HR-TEM image, (**d**) SAD pattern, (**e**) XRD pattern, (**f**) EDS pattern, (**g**) XPS pattern, and (**h**) Raman spectrum pattern.

TEM image and SEM image of CNTs (Fig. 1a,b) shows that the inner/outer diameter of the prepared hollow CNTs was approximately 15/60 nm, respectively. From HR-TEM image of CNTs (Fig. 1c), the interlayer spacing on the wall was 0.34 nm consisting with the distance of reported CNTs lattice^23^. The SAD pattern (Fig. 1d) further confirmed that the synthesized CNTs were well crystalline. The interlayer spacing of the CNTs was calculated using the Debye-Scherrer formula: D = Kλ/(β cosθ), where K is the Sherrer constant (0.89 in this study), λ is the X-ray wave length, β is the peak width at half-maximum, and θ is the Bragg diffraction angle. The XRD peaks meant that the CNTs had an interlayer spacing of about 0.34 nm in accordance with the result of HR-TEM image. As shown in XRD pattern (Fig. 1e), the main peak around 2θ = 25.8° (JCPDF 25-0284) represented the graphitic peak in accordance with C of the CNTs as well as a good crystallinity of the CNTs^24^. EDS pattern is shown in Fig. 1f, the peaks of the C, O, and Cu were clearly observed. From the XPS pattern (Fig. 1g), there were three main peaks (located in 284.69 eV, 286.52 eV, and 288.82 eV) which were represented to aromatic rings of N connected sp^2^ hybridized C atoms (N-C=N, C-OH, and N=C-N_2_)^25^,^26^. Because of no Cu peak in XRD image and from the XPS pattern (Fig. 1h), the Cu was caused by the conductive adhesive for affixing the samples. Furthermore, the same situations also appear in the EDS spectra and XPS patterns of the GA and CNSs. Raman spectrum (Fig. 1g) had two obvious vibration peaks consisting of 1360 cm^−1^ (D- peak) and 1580 cm^−1^ (G- peak). The D- peak and G- peak were reported to relate to the disordered structure (sp^3^) and the ordered structure (sp^2^) of carbon-based materials, respectively^27^. The inverse of the ID/IG intensity ratio between the G- and D- bands is a usual measure of graphitic ordering and may also indicate approximate layer size in the hexagonal plane, La = 44(ID/IG)^1/2 ^28,29. In this work, the ID/IG ratio of the CNTs was about 0.78. The result indicated that a potential application of synthesized CNTs was catalysis due to the defects in the walls of them. Based on the above results, it is clearly that the CNTs were produced using grasses as raw materials.

### Structure and Morphology of GA

**Figure 2:**
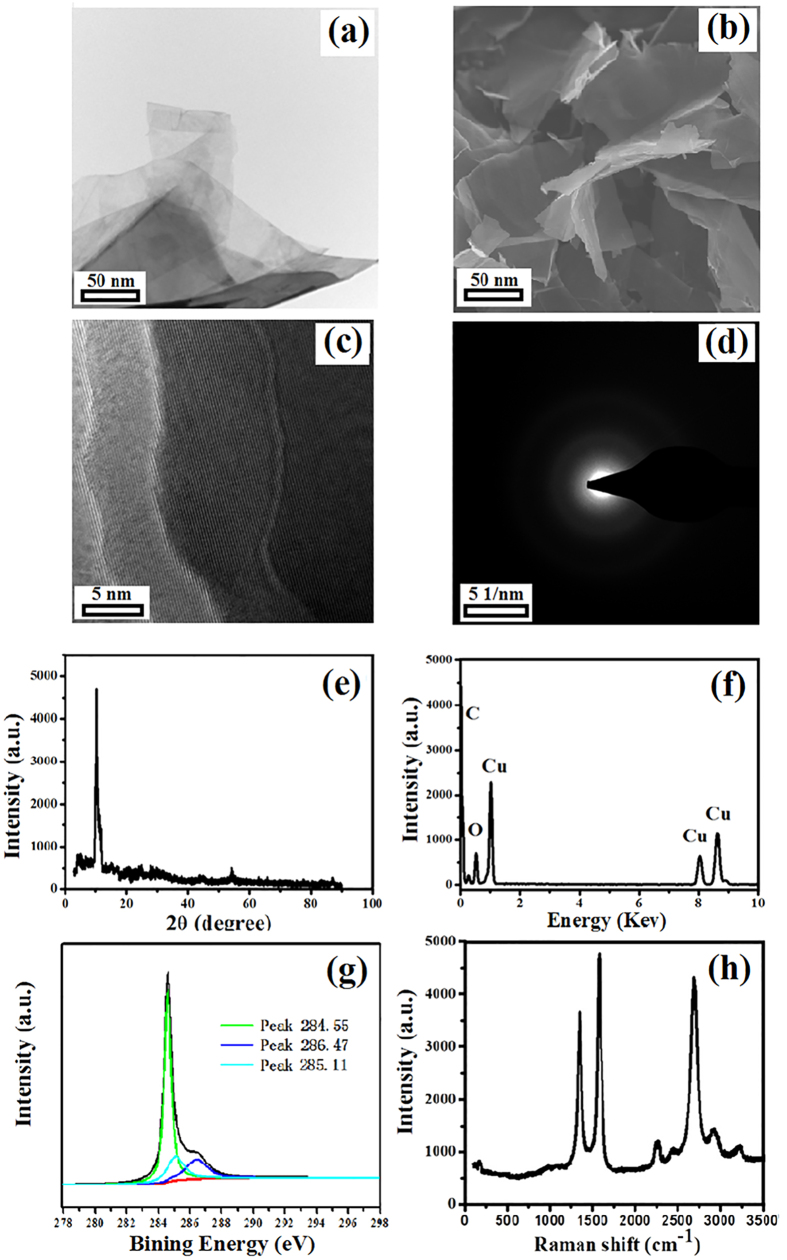
Characterization of the prepared GA from grasses: (**a**) TEM image, (**b**) SEM image, (**c**) HR-TEM image, (**d**) SAD pattern, (**e**) XRD pattern, (**f**) EDS pattern, (**g**) XPS pattern, and (**h**) Raman spectrum pattern.

TEM image and SEM image (Fig. 2a,b) of prepared GA from grasses shows a sheet like morphology and the wrinkles indicative of the flexibility of GA sheets as a consequence of the few-layer structure. As shown in HR-TEM image of GA (Fig. 2c), the interlayer spacing on the wall was 0.36 nm. Different from that of the CNTs, the SAD pattern (Fig. 2d) also indicated that there were a few defects on the surface of GA. Compared with XRD pattern of the CNTs, the main peak of prepared GA (Fig. 2e) ranged from 25.8° to around 10.4°. The result meant the interlayer distance was 8.7 Å and corresponding to the distance of between GA sheets. It also indicated the GA oxide nano-ribbons were prepared successfully through the unzipping of CNTs^30^,^31^,^32^. The interlayer spacing of the GA was also calculated by the Debye-Scherrer formula, and the result showed that interlayer spacing of them was about 0.36 nm. The same as EDS pattern of the CNTs, the Cu peaks in Fig. 2f were also contributed to the using of conductive adhesive. From the XPS pattern (Fig. 2g), there were three main peaks (located in 284.55 eV, 286.47 eV, and 285.11 eV) which were represented to the aromatic ring and N linked to sp^2^ hybridized C atoms (N-C=N, C-OH, and C-C on the surface of GA). As shown in Raman spectrum (Fig. 2h), the D- peak and G- peak were lower than the CNTs and the position and intensity of 2D bands at about 2700 cm^−1^ also provided an indication of the few-layer configuration of the GA^33^. The ID/IG ratio of the GA was about 0.39. The result indicated that the defects on the surface of GA were lesser than the synthesized CNTs. Therefore, it is clearly that the GA was produced using grasses as raw materials.

### Structure and Morphology of CNSs

**Figure 3:**
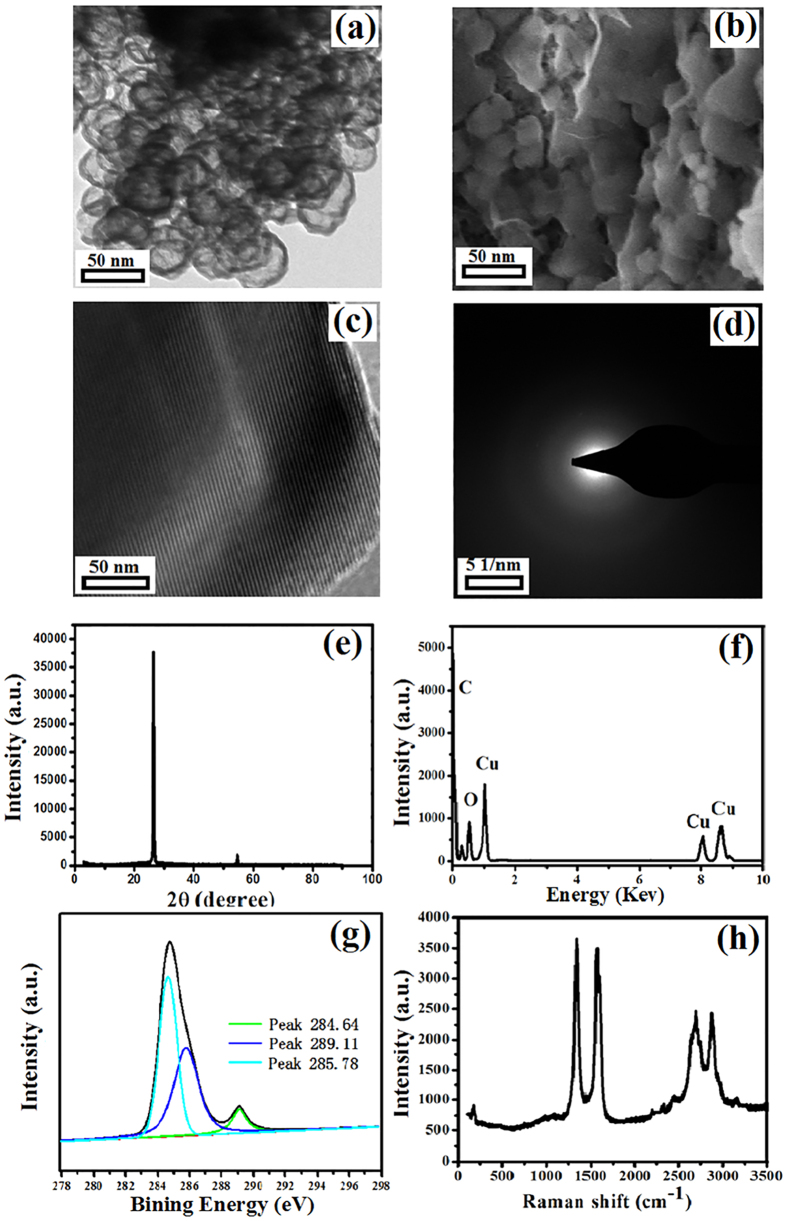
Characterization of the prepared CNSs from grasses: (**a**) TEM image, (**b**) SEM image, (**c**) HR-TEM image, (**d**) SAD pattern, (**e**) XRD pattern, (**f**) EDS pattern, (**g**) XPS pattern, and (**h**) Raman spectrum pattern.

TEM image and SEM image (Fig. 3a,b) demonstrates a sphere morphology and the diameter of prepared hollow CNSs was 40 nm. From HR-TEM image of CNSs (Fig. 3c), the interlayer spacing on the surface was 0.44 nm. Consisted with that of the GA, the SAD pattern (Fig. 3d) indicated that there were also some defects on the surface of the CNSs. As shown in XRD pattern (Fig. 3e) of the CNSs, the obvious diffraction peaks around 26.4° and 53.7° were corresponding to the (002) and (004) reflection of the graphitic peaks^34^. The interlayer spacing of the CNSs was also calculated by the Debye-Scherrer formula, and the result indicated that interlayer spacing of them was about 0.44 nm. Just like the CNTs and GA, the Cu peaks in Fig. 3f were also caused by the conductive adhesive. From the XPS pattern (Fig. 3g), there were three main peaks (located in 284.69 eV, 289.11 eV, 285.78 eV) which were represented to aromatic rings of N connected sp^2^ hybridized C atoms (N-C=N and C-OH) and sp^3^ hybridized C atoms (N=C-N_2_). The D- peak and G- peak are also observed in Raman spectrum (Fig. 3h). The ID/IG ratio of the CNSs was about 1.02. The result indicated that the defects on the surface of them were more than the synthesized CNTs and GA. The result indicated that a potential application of synthesized CNSs was adsorption due to the abundant defects on the surface. Thus, it is clearly that the CNSs were produced using grasses as raw materials.

In addition, the elementary compositions (C, O, and N) of CNMs are shown in the Table 1.

### Mechanism of Preparation of CNMs

Two different mechanisms were proposed for the CNTs formation by pyrolysis of natural cellulosic materials. Kang *et al.* firstly reported the formation of CNTs from grass and hypothesized that their formation was due to the oxidation of vascular bundles and the contraction of the tubular structures of vascular bundles afterwards^22^. Whereas, Goodell *et al.* proposed another hypothesis^20^: the cellulose microfibril arrangement occurring naturally within plant walls aided in the formation of CNTs when plant fiber was carbonized in multiple oxidation processes. Though the formation mechanisms were not confirmed, the experimental processes were similar for oxidation and carbonization. In addition, both of them suggested the importance of presence of O_2_ and tubular structures formed in purified lignin and cellulose. Although, the theories were also be confirmed by our previous work^35^,^36^, the effect about hydrolysis of cellulose and lignin on the synthesis process was not taken into account. During Kang’s approach^22^, water is lost, first from that absorbed by the cellulose and then by *β*-elimination from the cellulose hydroxyls, which makes the tubular structures contract and realizes the formation of C=C. Simultaneously, the oxygen makes the pyrolytic reactions of the vascular bundles more rapid. The complex chemistry of the C–O–H system is also helpful in the synthesis of nano-structured carbon. In our experiment, the oxidation and carbonization of HNO_3_ and H_2_SO_4_ were similar to above pyrolysis methods. Nevertheless, it was the fact that the CNTs could be also primarily prepared owing to the hydrolysis of cellulose and lignin under acidic condition. With the presence of more oxidants in this work, the prepared CNTs could farther be translated to other CNMs. The cellulose is simpleness composed of polymer of β-(1–4) D-glucopyranosyl^37^. However, the composition lignin is complexity. The portion of lignin shows that there were lots of monomers (mostly coniferyl alcohol, sinapyl alcohol, and p-coumaryl alcohol) and atoms of carbon, hydrogen, and oxygen^38^. These portions were incorporated into lignin in the form of the p-hydroxyl phenyl propanoids, guaiacyl propane, and syringyl propane, respectively^39^. The grasses have most guaiacyl propane, and all lignin contains small amounts of incomplete or modified monolignols^40^,^41^. Compared with the lignin, the cellulose must be hydrolyzed much easier and faster. Furthermore, the hydrolyzate of cellulose was D-glucose (following equation) which dissolved easily in water. According to literature, only 6.3% of acidic hydrolysis product from lignin was dissolved^42^. The insoluble lignin was oxidated and carbonized to form carbon skeleton of the CNTs. Based on above discussion, the difference of hydrolysis rate of between cellulose and lignin and solubility of hydrolyzate from them played important roles in the formation of carbon skeletons of the CNTs.


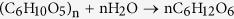


In general, the CNTs could be viewed as a roll-up sheet of the GA. However, according to recent literature, the GA could be prepared through opening the CNTs along the axial by oxidation and the CNSs could be prepared through rolling-up sheet of the GA^42^. The oxidative chemical unzipping of CNTs using acid and oxidant offered a unique way for the bulk production of GA owing to oxidation process open up the end caps of CNTs end and cutting them longitudinally^43^,^44^. There were lots of defects on the surface in walls of prepared CNTs. With H_2_O_2_/HNO_3_/H_2_SO_4_, the bond-angle strain induced the defects to form hole (or tear if originating from the end of the CNTs). Once an opening has been initiated, its further opening was enhanced relative to an unopened tube or to an uninitiated site on the same tube. Hence, relief of the bond-angle strain of CNTs opens to the GA^45^. Hence, CNSs were overlapped on the edges of GA and linked together like winding chains^46^.

**Figure 4:**
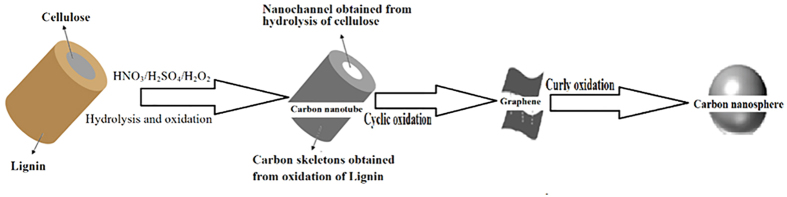
Formation schematic of the CNMs from grasses.

In the current experiment, with H_2_O_2_/HNO_3_/H_2_SO_4_, the CNTs were firstly prepared from grasses at 300 °C by hydrolysis and oxidization, the CNTs were secondly opened to form the GA by oxidization, and the GA was lastly rolled-up to form the CNSs by oxidization. The formation schematic of CNMs from grasses is presented in Fig. 4.

### Adsorption of 2-CP

**Figure 5:**
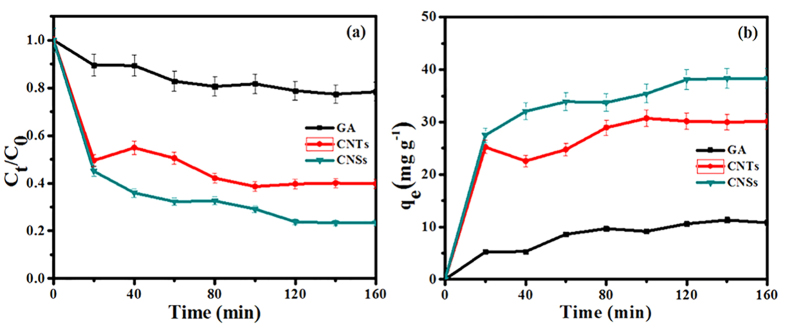
Adsorption equilibrium time of the CNMs for 2-CP: (**a**) efficiencies, (**b**) capacities.

The adsorption equilibration time curves of the CNTs, GA, and CNSs to 2-CP are given in Fig. 5. All complete equilibration times of adsorption of 2-CP were within 120 min, and approximately 60.35%, 20.12%, and 76.22% of 2-CP (5 mg L^−1^) were adsorbed on the CNTs, GA, and CNSs (Fig. 5a), respectively. The equilibrium adsorption capacities of three types of the CNMs to 2-CP were in according to the order as CNSs > CNTs > GA. As the result, the CNSs was more suitable for removal 2-CP in water as adsorbent than the CNTs and GA.

pH has an important impact on the adsorption of the CNTs, GA, and CNSs to 2-CP. The pKa of 2-CP is 8.56^47^, so that 2-CP exists as a phenolate (2-Cl-C_6_H_4_O^−^) when the pH is >8.56, and as molecular (2-Cl-C_6_H_4_OH) when the pH is <8.56. The pH of test 2-CP aqueous solution was 6.3, most of them was presented as molecular state. The octanol-water partition coefficient (log k_ow_) was 2.15 for 2-CP, it meant 2-CP was particularly soluble in water and not expected to be adsorbed to a greater extent^48^.

However, up to approximately 30.18 mg g^−1^ and 38.11 mg g^−1^ (conversion according to Fig. 5b) of adsorbing capacities of the CNTs and CNSs for 2-CP (5 mg L^−1^) reached until equilibration took place. Although the three types of the CNMs were prepared from the same carbon source, materials, and chemicals, their adsorption properties to 2-CP were different. The adsorption of 2-CP can also be correlated with the structures and morphologies of the CNTs, GA, and CNSs. As shown in Table 2, BET results indicated that the prepared CNTs, GA, and CNSs had specific surface areas of 249.43 m^2 ^g^−1^, 208.66 m^2 ^g^−1^, and 327.84 m^2 ^g^−1^, respectively, meaning that they were potential to be used as adsorbents. The BJH surface area of pores, BJH mesopore value, and BJH average mesopore diameter were 290.01 m^2 ^g^−1^, 0.6121 cm^3 ^g^−1^, and 6.90 nm for the CNTs, 175.32 m^2 ^g^−1^, 0.4735 cm^3 ^g^−1^, and 6.90 nm for the GA, and 395.06 m^2 ^g^−1^, 0.7470 cm^3 ^g^−1^, and 8.13 nm for the CNSs. In our work, the surface area for CNTs was greater than GA, due to the weak interaction between GA sheets and the broken sheet structure of them. In addition, the main peak (XRD) of GA ranged from 25.8° to around 10.4°, the increase of interplanar spacing also indicated that the structure of GA has changed and the GA layers had been loosed^49^. So, BET surface areas and pores of CNTs were appreciable greater than GA. Based on the results, the specific surface area of CNSs was higher than both CNTs and GA. The prepared CNTs and CNSs from grasses exhibited a higher adsorption activity than the GA, while the CNTs was lower.

**Figure 6:**
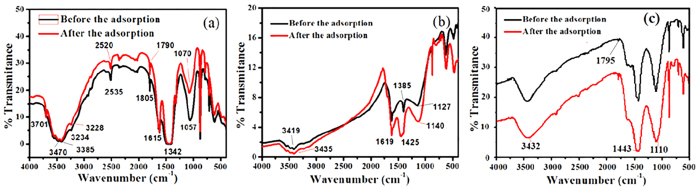
FTIR spectra of the CNTs, GA, and CNSs before and after the adsorption of 2-CP.

To justify the observed behavior, the functional groups on the prepared the CNMs should be contemplated. From Fig. 6, the groups -OH (peaks at 3701 cm^−1^, 3470 cm^−1^, 3385 cm^−1^, and 3234 cm^−1^), C≡C (peak at 2535 cm^−1^), C=O (peak at 1805 cm^−1^), and C=O-OH (peak at 1615 cm^−1^) existed on the surface of the CNTs; the groups of -OH (peaks at 3419 cm^−1^ and 1127 cm^−1^) and C=O-OH (peak at 1619 cm^−1^) existed on the surface of the GA; the groups of -OH (peak at 3432 cm^−1^and 1110 cm^−1^), C=O (peak at 1795 cm^−1^), and aromatic ring (peak at 1443 cm^−1^) existed on the surface of the CNSs. Furthermore, after the adsorption procedure, all above functional groups shifted to lower wave numbers indicated that they played a role in the adsorption of 2-CP. Based on the analysis for the main characteristic peak positions and intensities, the amount of active groups on three types of the CNMs to 2-CP were in the order of CNSs > CNTs > GA. In brief, the different adsorption properties of prepared the CNMs to 2-CP were caused by the differences of structures and morphologies.

**Figure 7:**
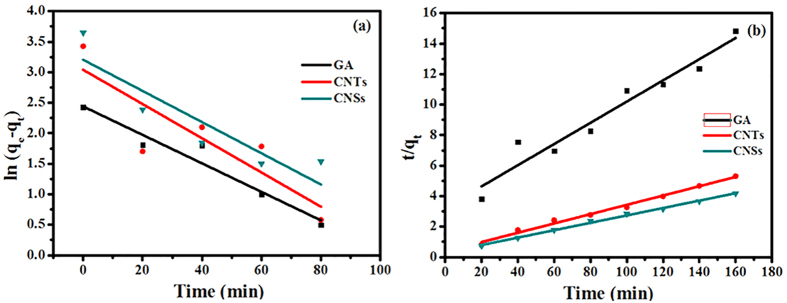
Adsorption kinetics of the CNMs for 2-CP: (**a**) pseudo-first-order kinetics, (**b**) pseudo-second-order kinetics.

Adsorption kinetics of 2-CP on the CNTs, GA, and CNSs is shown in Fig. 7 and Table 3. The correlation coefficients (*R*^2^) of pseudo-first-order kinetics were in a range of 0.7566–0.9479 and *R*^2^ of pseudo-second-order kinetics were in a range of 0.9456−0.9963. The results demonstrated that the adsorption kinetics data of three types of the prepared CNMs were more in agreement to the pseudo-second-order model, the chemical adsorptions occurred of them for 2-CP and adsorption capacities were proportional to the numbers of active sites on them^50^.

**Figure 8:**
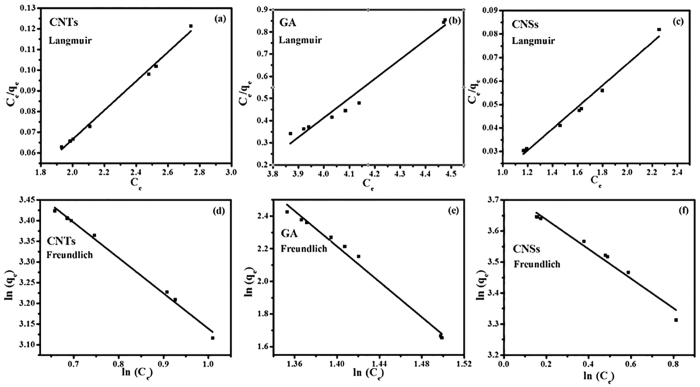
Adsorption isotherms of the CNMs for 2-CP: (**a–c**) Langmuir isotherm of the CNTs, GA, and CNSs, (**d–f**) Freundlich isotherm of the CNTs, GA, and CNSs.

The adsorption isotherms and adsorption parameters (q_max_, K_L_, K_F_, n, and *R*^2^) of the CNMs for 2-CP are shown in Fig. 8 and Table 4. The equilibrium data were fitted to the Langmuir and Freund lich illustrating the effect of initial 2-CP concentration on adsorption equilibrium at studied temperatures. Considering the goodness-of-fit (*R*^2^), the Freund lich model was more appropriate for the adsorption of 2-CP (0.9728 < *R*^2^ < 0.9953) than the Langmuir models. The equilibrium data were also well fitted to the Langmuir equation but with slightly smaller correlation coefficients (0.9725 < *R*^2^ < 0.9952). From the results, the adsorption processes of three types of the CNMs for 2-CP took place on heterogeneous surfaces and adsorption capacity was related to the concentration of 2-CP at equilibrium^51^.

### Photocatalytic Degradation of 2-CP

**Figure 9:**
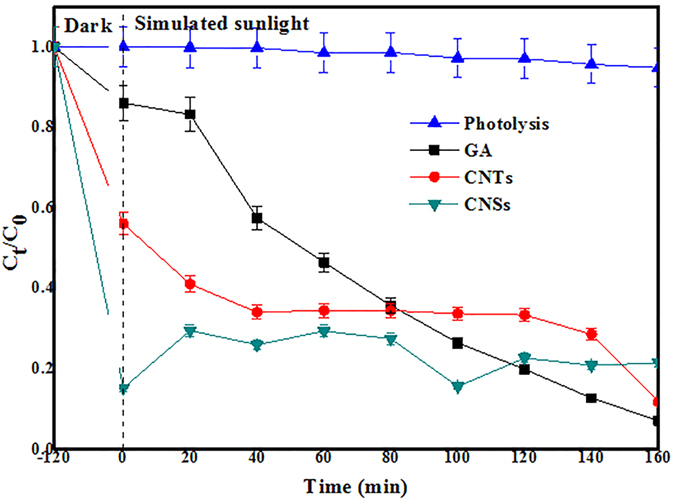
Degradation of 2-CP by photolysis and photocatalysis.

The typical time courses of photolysis and photocatalytic degradation for 2-CP are shown in Fig. 9. The high removal rates (C_t_/C_o_) of 2-CP after 160 min irradiation for the CNTs, GA, and CNSs were about 88.23%, 92.90%, and 79.64%, respectively. However, the removal rates were contributed from three origins: (1) photolysis, (2) adsorption by the CNMs, and (3) photocatalytic degradation by the CNMs. The 2-CP could hardly be degraded in the control experiment (photolysis), the average degradation of photolysis for 2-CP (5 mg L^−1^) was only 6.11% with simulated sunlight irradiation for 160 min. The adsorption efficiencies of the CNTs, GA, and CNSs for 2-CP were approximately 44.91%, 14.93%, and 84.80%. Hence, photocatalytic degradation (approximately equal that removal efficiency minus efficiency of adsorption and photolysis) of 2-CP by the CNTs, GA, and CNSs materials were 37.21%, 72.79%, and −11.27%. Herein, the prepared the GA and CNTs from grasses exhibited a higher photocatalytic activity than the CNSs, while the CNTs was lower.

Compared with the results of adsorption, the adsorption efficiency of the CNSs for 2-CP changed from 76.22% (adsorption test) to 84.80% (photocatalytic degradation test), and the removal efficiency of the CNSs for 2-CP altered from 84.80% (begin of photocatalytic degradation) to 79.64% (end of photocatalytic degradation). The negative values were caused by the easy aggregation of the CNSs with the time increasing which was observed frequently in the experiment. In our work, the aggregation of CNSs was observed obviously, owing to the specific surface area and strong van der Waals force of them. The aggregation of CNSs decreased the specific surface area, free energy of system, adsorptive sites, and activity. As a result, the aggregation of CNSs was attributed to the negative value of photocatalytic degradation for CNSs and led to the release of the adsorbed 2-CP. Moreover, the retention time of analysis by HPLC (at 254 nm) for 2-CP after photocatalytic degradation is demonstrated in Table 5. Based on the above discussion and Table 4, 2-CP was completely degraded to CO_2_ and H_2_O by photocatalysis of the GA, and the intermediate product existed steadily in the process of photocatalytic degradation of the CNTs for 2-CP, the removal of the CNSs to 2-CP was mainly due to the adsorption.

### Mechanism of 2-CP Photodegradation

**Figure 10:**
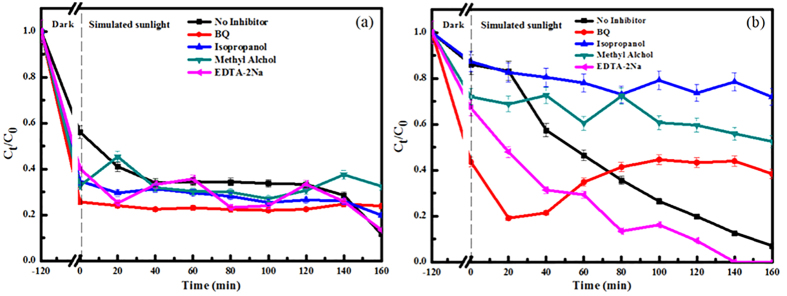
Plotted degradation curves of: (**a**) the CNTs and (**b**) the GA for 2-CP with additions of scavenger.

Photodegradation of 2-CP in the presence of reactive oxygen species (ROS) could be caused by the generation of ·OH, or O_2_^·−^, or both^52^. To find out the possible involvement of these ROS caused by the CNTs and GA in the degradation of 2-CP, photo-irradiation experiments in the presence of the BQ, isopropanol, methyl alchol, and the EDTA-2Na were carried out (Fig. 10), respectively. Because of no significant effect on the photocatalytic degradation of the CNSs for 2-CP, the experiment of radical scavengers for photodegradation of 2-CP by the CNSs were not carried out.

As shown in Fig. 10, compared with no inhibitor (43.32%), the degradation efficiencies (approximately equal removal efficiency minus adsorption efficiency) of 2-CP after 160 min were 0.13% for BQ, 14.88% for isopropanol, 1.79% for methyl alchol, 26.73% for the EDTA-2Na. In addition, the tendencies of removal efficiencies of CNTs for 2-CP were not obviously changed with the addition of the BQ. The BQ inhibited the photodegradation by scavenging O_2_^·−^ which is a highly reactive intermediate of oxidative active species^53^. The BQ was able to inhibit the photodegradation of the GA for 2-CP, suggesting that the photooxidation of 2-CP was mainly initiated by O_2_^·−^. ·OH is an extremely potent oxidizing agent that unselectively oxidizes various kinds of organic pollutants and with high rate constants^54^,^55^. The degradation efficiencies was decreased to 14.88% with addition of isopropanol, which meant ·OH also partly effected the photocatalytic degradation of the CNTs for 2-CP. Moreover, the majority of ·OH radicals could be converted into HO_2_·/O_2_^·−^ by methyl alchol^56^. If HO_2_·/O_2_^·−^ were an effective oxidant to 2-CP, the degradation would be greatly increased, which was inconsistent with the results shown in Fig. 10a. The degradation efficiency of the CNTs for 2-CP with EDTA-2Na was corresponded closely to that without inhibitor. EDTA-2Na is a hole scavenger^57^, the results meant h^+^ did not play an important role in the photocatalytic degradation of the CNTs for 2-CP. In addition, the fluctuation of the degradation efficiency was caused by the increasing on separation of photon-generated carrier and decreasing on the generation of ·OH from the reaction of hole and H_2_O after holes being inhibited. From the above discussion, O_2_^·−^ and ·OH that generated in the photodegradation of the CNTs were responsible for 2-CP decomposition, while the O_2_^·−^ played an important role.

From Fig. 10b, compared with no inhibitor (77.97%), the degradation efficiency of 2-CP was 5.18% for the BQ, 15.45% for isopropanol, and 19.50% for methyl alchol. Furthermore, 2-CP was completely photocatalytic degraded by the combine with simulated sunlight irradiation and the prepared GA in 140 min after being added the EDTA-2Na into 2-CP aqueous solution.

Although the BQ inhibited the photo degradation by scavenging O_2_^·−^, the degradation efficiencies of 2-CP increased at the beginning of the experiment owing to the increasing of generation of ·OH from holes and H_2_O, separation of photon-generated carrier, and quantum efficiency. With the addition of methyl alchol, the degradation of the GA for 2-CP was inhibited, indicating the effective oxidant to 2-CP was not O_2_^·−^, but HO_2_·. The degradation efficiency of the GA for 2-CP with addition of the EDTA-2Na was higher than control, which implied h^+^ played less role on the photocatalytic degradation of the GA for 2-CP. Moreover, the enhanced photocatalytic property was caused by separation of photon-generated carrier and generation of mass O_2_^·−^ from the reaction of photo electron with O_2_ in the 2-CP/GA/EDTA-2Na system. The degradation efficiencies were lower than control at any time in the experiment with addition of isopropanol. Based on the above discussion, ·OH played an important role in the photocatalytic degradation of the GA for 2-CP.

O_2_^·−^, H_2_O_2_, ·OH, HO_2_·, ROO·, RO·, etc. are ROS, and the oxidizability of ·OH is the strongest. An O_2_^·−^ radical finally reacted with H^+^ in the water to produce HO_2_· before generating two ·OH radicals^58^. In other words, if a ROS radical (except ·OH) can generate more ·OH, its oxidizability will be greatly improved. So, the ROS oxidizability standard coefficient (ROSosc, ROSosc of ·OH is 1) and the ROS oxidizability actual coefficient of a material (ROSoac, when pollutants was just completely oxidized to CO_2_ and H_2_O by ·OH from the material, the ROSoac is 1) were defined as follows:


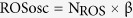


N_ROS_ is the number of ·OH can be produced from a ROS radical (O_2_^·−^ is 2), β is the rate of actual oxidizability of ·OH to ROS.


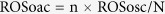


n is the number of generated actually ROS radicals from the material, N is the number of demand ·OH for oxidizing the pollutant to CO_2_ and H_2_O.

**Figure 11:**
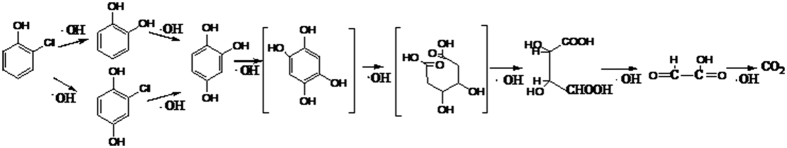
Possible reaction pathway involving ·OH radicals.

However, the 2-CP was incompletion photocatalytic degraded by the CNTs, it meant that ROSoac of the combine with simulated sunlight irradiation and the CNTs were less than that from the combine with irradiation and the GA. From the results, the stable intermediate product found in photodegradation of the CNTs for 2-CP can be derived from the reaction of O_2_^·−^ and 2-CP or ·OH and 2-CP. The possible reaction pathway involving ·OH radicals is shown in Fig. 11. According to the literature, 2-CP may lead to the formation of 2-Cl-BQ with the presence of O_2_^59^. Furthermore, the intermediate product was confirmed to be the 2-Cl-BQ by HPLC with standard solution (shown in Table 4). As a consequence, the photocatalytic degradation of CNTs for 2-CP could lead to the increasing toxicity in the water^60^. GA was more suitable for removal 2-CP in water as photocatalyst than CNTs and CNSs.

## Conclusion

The CNMs (CNTs, GA, and CNSs) were prepared respectively using the same grass as the sole carbon resource through a simple solvothermal process, as well as the 2-CP in water could be effectively removed by them. However, the mechanisms of formation of the CNTs, GA, and CNSs from grasses and reaction pathways of removal 2-CP were different. The CNTs were primarily prepared owing to the hydrolysis of cellulose and lignin solubility of hydrolyzate from them with H_2_O_2_/HNO_3_/H_2_SO_4_; the CNTs were secondly opened to form the GA by oxidization; and the GA was lastly rolled-up to form the CNSs by oxidization. The CNSs was more suitable for removal 2-CP in water as adsorbent than the CNTs and GA. The adsorption of three types of prepared CNMs for 2-CP was chemical adsorption and took place on heterogeneous surfaces. The GA was more suitable for removal 2-CP in water as photocatalyst than the CNTs and CNSs. O_2_^·−^ and ·OH played an important role in the photocatalytic degradation of the CNTs and GA for 2-CP, respectively. However, the photocatalytic degradation of the CNTs for 2-CP could lead to the increasing toxicity of water.

## Experimental Section

### Materials and Chemicals

The species of grasses used for preparing the CNMs was *Festuca arundinace* and collected randomly from the campus of Northeast Normal University. 2-CP 97% + and 2-chloro-1,4-benzoquinone (2-Cl-BQ) 99% + were obtained from Tianjing Tiantai Fine Chemicals Co., Ltd. and used without further purification. H_2_O_2_ (30%), HNO_3_ (69.2%), and H_2_SO_4_ (98%) were provided by Tianjing Fucheng Chemical Reagent Factory. Only second distilled water was used in current study.

### Preparation and Characterization of the CNMs

10 g collected grasses were clipped and mixed with 10 ml H_2_O_2_, 5 ml HNO_3_, and 5 ml H_2_SO_4_ in reactors after particles adhering to the surface of them had been removed with water, respectively. The reactor was corrosion resisting stainless steel-lined reaction kettle, and the volume was 100 ml. Three reactors were respectively heated with the same heating procedure (10 °C min^−1^) to 300 °C for synthesis of the CNTs, 400 °C for synthesis of the GA, and 500 °C for synthesis of the CNSs, and then kept at the temperature for 60 min. The natural cooling reaction solutions were filtered 3–5 times with glass filter paper (0.45 μm). Afterwards, the residues containing the CNTs, GA, and CNSs were collected, washed 3 times with ethanol, and dried at 105 °C for 2 h.

The prepared CNMs were characterized by the methods as follows: transmission electron microscopy (TEM) images and high-resolution TEM (HR-TEM) images were performed using a Philips EM208 working at 20 kV; energy dispersive spectra (EDS) were obtained using an Oxford EDX system attached to TEM; selected area diffraction (SAD) patterns were taken on HR-TEM attached to TEM; X-ray diffraction (XRD) patterns were obtained on a Rigaku D-max C III (Ni-filtered Al Kα radiation); Raman spectra were obtained using a micro-Raman spectrometer (Nicolet Almega XR) with a 473 nm laser as an excitation source; Fourier transform infrared (FTIR) spectra (4,000–400 cm^−1^) were performed using a Nexus 670 FTIR spectrometer (Thermo Nicolet, Madison) equipped with a KBr beam splitter (KBr, FTIR grade); surface areas were determined by a N_2_ Brunauer-Emmett-Teller (BET) gas adsorption-desorption method at 77 K (Micromeritics ASAP-2000, USA); mesopore size distributions and volumes were obtained from the N_2_ desorption isotherms by Barrett-Joyner-Halenda (BJH) method; the 2-CP concentrations were monitored with HPLC equipped with a Dionex C-18 reversed-phase LC column. The eluent was a ternary mixture of water (containing 1% v/v acetic acid) and acetonitrile (40:60), pumped at a rate of 0.8 mL min^−1^, retention times 15 min.

### Adsorption and Photocatalysis

The first step in the adsorption experiment was the measurement of the adsorption capacities of 2-CP by the CNMs in batch adsorption tests. For determination of the adsorption isotherms, 10 mg prepared CNMs were contacted with fixed concentrations of 100 mL 2-CP aqueous solution (5 mg L^−1^ and pH at 6.3) and the mixture was stirred (150 rpm and 25 °C) until reaching equilibrium. The time required for reaching the equilibrium of 2-CP concentration was the equilibrium time for adsorption. At different time intervals, the supernatant was collected and centrifuged at 1,500 rpm for 20 min in the centrifuge. Afterwards, the 2-CP concentration was determined by HPLC after the supernatant was filtered through 0.45 μm Millipore cellulose acetate membrane to remove the CNMs. In this work, the equilibrium adsorption capacity of the adsorbent was calculated, the Langmuir and Freundlich were employed to describe the adsorption, and both the pseudo-first-order kinetic model and the pseudo-second-order kinetic model were used to model the kinetic data.

Photodegradation experiments of 2-CP were conducted in the quartz reactor. 10 mg prepared CNMs were added into 100 mL 2-CP (5 mg L^−1^ and pH at 6.3) aqueous solution. To ensure adsorption-desorption equilibrium of 2-CP on the CNMs, the mixtures were stirred (150 rpm and 25 °C) in dark for 120 min (reaching the equilibrium) in quartz reactors. It was then stirred under irradiation with a xenon lamp (30 W), which served as a simulated sunlight irradiation source with a cut-off filter. There actor was placed and irradiated within the distance of 15 cm from the lamp. At 20 min intervals, 2 mL aqueous solution was withdrawn from the reaction system and separated by centrifugation to obtain a supernatant liquid free from the CNMs. During the photocatalytic decomposition process, the separated samples were analyzed by HPLC after the supernatant was filtered through 0.45 μm Millipore cellulose acetate membrane to remove the CNMs. The control experiment (photolysis) of 2-CP without the CNMs was also investigated.

To elucidate the CNTs-sensitized and GA-sensitized photodegradation mechanisms of 2-CP, the 0.01081 g 4-benzoquinone (BQ, for O_2_^·−^), 0.1 mL isopropanol (99.7%, for ·OH), and 0.0037 g ethylenediaminetetraacetic acid disodium salt (EDTA-2Na, for hole), used as the radical scavenger, were added into the aqueous solution of 2-CP with the CNMs. Furthermore, 0.4 mL methyl alchol (99.5%, for O_2_^·−^ and ·OH_2_) was used as the transforming agent for ·OH to HO_2_·/O_2_^·−^ 61. The procedures were according to the photodegradation experiment. The control (photolysis) samples contained 2-CP only.

### Statistical Analysis

In this work, three replicates were carried out. The linear form of kinetic and isotherm equations were applied to the data of adsorption and photocatalysis. The correlation coefficient (*R*), the level of significance (*p*-value), and the standard error (SE) were used to identify the best-fit model of adsorption and photocatalysis.

## Additional Information

**How to cite this article**: Ma, Y. *et al.* Comparative Study of Carbon Materials Synthesized “Greenly” for 2-CP Removal. *Sci. Rep.* **6**, 29167; doi: 10.1038/srep29167 (2016).

## Figures and Tables

**Table 1:** Atomic percentages of C, O, and N in CNMs.

Carbon nano materials	C	O	N
CNTs	66.57%	30.56%	2.87%
GA	76.73%	21.04%	2.23%
CNSs	73.43%	23.62%	2.95%

**Table 2:** Surface area and pore structure of CNTs, GA, and CNSs obtained from grasses.

Carbon nano materials	BET surface area (m^2^g^−1^)	BJH surface area of pores (m^2^ g^−1^)	BJH mesopore value (cm^3^ g^−1^)	BJH average mesopore diameter (nm)
CNTs	249.43	290.01	0.6121	6.90
GA	208.66	175.32	0.4735	5.47
CNSs	327.84	395.06	0.7470	8.13

**Table 3:** Adsorption kinetics model of 2-CP on carbon nano materials.

Carbon nano materials	pseudo-first-order kinetic				pseudo-second-order kinetic			
	Formula	q_1_	K_1_	*R*^2^	Formula	q_2_	K_2_	*R*^2^
CNTs	y = −0.028x + 3.039	20.8843	0.028	0.7566	y = 0.0305x + 0.3756	32.7869	0.0025	0.9903
GA	y = −0.0233x + 2.4394	11.4662	0.0233	0.9479	y = 0.0695x + 3.245	14.3885	0.0015	0.9456
CNSs	y = −0.0255x + 3.2039	24.6284	0.0255	0.8192	y = 0.0243x + 0.3074	41.1523	0.0019	0.9963

**Table 4:** Langmuir and Freundlich models of carbon nano materials for 2-CP.

Carbon nano materials	Langmuir				Freundlich			
	q_max_	K_L_	*R*^*2*^	Formula	n	K_F_	*R*^*2*^	Formula
CNTs	14.2045	−0.9475	0.9952	y = 0.0704x − 0.0743	−0.8562	54.315	0.9953	y = −0.8562x + 3.9948
GA	1.1382	−0.2833	0.9725	y = 0.8786x − 3.1015	−5.4216	18113.27	0.9883	y = −5.4216x + 9.8044
CNSs	1.1382	−0.2833	0.9725	y = 0.8786x − 3.1015	−0.4762	41.7625	0.9728	y = −0.4762x + 3.732

q_max_ is the maximum adsorption capacity (mg g^−1^), K_L_ is the Langmuir constant (L mg^−1^), K_F_ is the adsorption equilibrium constant (mg g^−1^ (mg L^−1^)^−n^), and n is a constant indicative of adsorption intensity, *R* is correlation coefficients.

**Table 5:** Retention time of analysis for the 2-CP.

Samples	Retention time (min)
2-CP	7.562
2-CP with CNTs	6.375 and 7.616
2-CP with GA	7.566
2-CP with CNSs	7.574
2-Cl-BQ	6.359
